# Pre-Service Teachers Learning to Teach English to Very Young Learners in Macau: Do Beliefs Trump Practice?

**DOI:** 10.3390/bs12020019

**Published:** 2022-01-23

**Authors:** Barry Lee Reynolds, Xuan Van Ha, Chen Ding, Xiaofang Zhang, Sylvia Liu, Xiaoyan Ma

**Affiliations:** 1 Faculty of Education, University of Macau, Macau SAR, China; zhzxfang@gmail.com (X.Z.); yb77105@connect.um.edu.mo (S.L.); MC04876@um.edu.mo (X.M.); 2 Centre for Cognitive and Brain Sciences, University of Macau, Macau SAR, China; 3 Department of Foreign Languages, Ha Tinh University, Ha Tinh 45000, Vietnam; xuan.havan@htu.edu.vn; 4 School of Foreign Studies, Hefei University of Technology, Hefei 230601, China; dingchen_conan@163.com

**Keywords:** teacher cognition, teacher beliefs, pre-service teachers, English, EFL, teacher education, kindergarten, pre-primary, Macau, microteaching

## Abstract

The global importance of English and therefore the teaching of the English language has made the English language curriculum an integral part of all levels of teacher education, including early childhood education. The purpose of this study was to first explore the beliefs about the teaching of English to very young learners held by pre-service pre-primary teachers in Macau and then to see whether these beliefs were reflected in their microteaching. Qualitative content analysis performed on the written reflections and transcriptions of the microteaching videos of 75 pre-service pre-primary teachers found that their beliefs about classroom practices, lesson planning, and English as a foreign language (EFL) learners and learning were the most predominant beliefs exhibited in their reflections and were evidenced in their microteaching. Less predominant, but still salient, were their beliefs about the goals of language learning, assessment, teaching, pedagogical knowledge, and content. No observable practices were found regarding the pre-service teachers’ beliefs about the role of teaching, learning to teach, microteaching, the self, the subject, hearsay, self-assessment, and schooling. The current study found that with only limited exposure to EFL teaching methodology from a single course, the pre-service pre-primary teachers were able to implement some of their beliefs about several important aspects of teaching English to very young learners.

## 1. Introduction

“Teacher beliefs” refers to a key construct in teacher cognition [1]. Teacher beliefs encompass a teacher’s knowledge of objects, people, events, and their relationships with one another; these beliefs affect teacher decision making [2] and their subsequent classroom interactions with students [3]. In the context of English language teaching (ELT), teacher beliefs involve the nature of language learning, effective language teaching, the relationship between content knowledge and pedagogical knowledge, the role of a language teacher, and attitudes toward language teacher education [4]. This construct is shaped by teachers’ prior experiences [5], including the teacher education curriculum or training teachers have received (e.g., knowledge, skills, and teaching practice), and the beliefs and practices of their teacher educators (e.g., feedback) [6].

While a range of factors have been explored as potential catalysts for change in teacher beliefs, teacher education, which provides both theoretical and practical training, has been one of the most investigated areas (e.g., [5]). Still, few studies have compared the beliefs held by English teachers to their actual practices (see [7] as one such attempt) and even fewer have looked at how the beliefs and practices of pre-service pre-primary English teachers align or misalign [3]. Previous research has shown that pre-service English as a foreign language (EFL) teachers tended to prioritize pedagogical content knowledge and that they were influenced by teacher education curricula that incorporated reflection [8] and exploratory teaching [9]. Still, to the best of our knowledge, there has yet to be a study that has explored whether the beliefs of pre-service pre-primary English teachers coincide with their practices when undergoing pedagogical training that incorporates reflective practice and exploratory teaching activities. The recent debate surrounding early childhood English language education in China (e.g., [10,11]) prompts the current case study and marks it as timely and relevant to the research context—the Macau special administrative region of China. In Macau, English maintains an important role in communication, media, education, international trade, and the gaming industry [12]. Conducting a case study can underscore the effect of a teacher education program’s impact on pre-service EFL teachers’ beliefs about teaching English within the Macau SAR. The results of this study have the potential for enhancing and increasing the EFL teacher training provided to early childhood pre-service teachers in the context of education in the Macau SAR. The following research question guided the case study: To what extent are pre-service pre-primary English teachers’ beliefs reflected in their microteaching?

## 2. Literature Review

### 2.1. Teacher Beliefs

In the last forty years, studies about teacher beliefs have made a significant contribution to our understanding of teachers and teaching [13]. In general, a “belief” has four common features, including that it “may be consciously or unconsciously held,” “is evaluative in that it is accepted as true by the individual,” “is imbued with emotive commitment,” and “serves as a guide to thought and behavior” ([13], p.186). In particular, teacher beliefs play an essential role in many aspects of teaching and learning, especially “beliefs about teachers performing tasks and the nature of knowledge” ([14], p. 2). Teacher beliefs have been shown to be influenced by a teacher’s personal experience [15,16], teaching experience [16], educational background [17], and colleague support [16]. Moreover, the literature has provided insights into teacher beliefs concerning teaching practice [18,19,20], teacher education [21,22,23,24], learning experience [25], curriculum design [14,26] parent involvement [27], learner autonomy [13], and corrective feedback [28,29]. Researchers have also suggested that there is a strong relationship between teacher beliefs and their teaching practice in EFL classrooms [1,13,30,31]. Specifically, what teachers actually do inside the classroom is determined by their held beliefs and then are further shaped by their desirability and feasibility of learning new skills [13,32].

### 2.2. Pre-Service English Teacher Beliefs

Teacher beliefs vary by teaching context and the stage in a teacher’s teaching career [3,33]. When compared to the teachers of other subjects, English teachers often use most of their time improving their English proficiency instead of acquiring pedagogical knowledge, which may result in the insufficient acquisition of practical teaching knowledge as well as underdeveloped teacher beliefs [3]. Not only will their teaching practice be affected by their latent beliefs, their students’ own beliefs about English learning may also be further influenced [34]. For example, unrealistic pre-service teacher beliefs that only value students’ time invested in learning as the key to improving language proficiency may lead to the students’ misconceptions about English learning; specifically, teachers might feel that the more time that students spend on learning, the higher level of proficiency they would achieve, which could obviously lead to the learners’ inefficiency and frustration [34,35]. Furthermore, differing from in-service teacher beliefs, pre-service teacher beliefs are often much easier to change because pre-service teachers lack teaching experience. When teachers have engaged in teaching for an extended time period, they start to follow certain teaching patterns, but pre-service teachers have not yet developed these patterns [36]. Thus, this may make pre-service teachers more susceptible to teacher education or interventions.

### 2.3. Change in Early Childhood Teacher Beliefs

Early childhood educators must master teaching techniques [37] that require both language and education competence [38]. Early childhood teachers’ ability to provide support in both the first and foreign languages is important for the young learners’ future language proficiency development [39,40]. Such skills are specialized and are encouraged by beliefs that differ from those of teachers of secondary or adult students [37,41]. McMahon et al. [42], for example, investigated the relationship between early childhood education teacher beliefs and children’s classroom involvement, finding a mediating effect of the teachers’ trust in an overarching theoretical principle. The early childhood teachers held the belief that children learn knowledge through active engagement in classroom activities and not through imitating adults [43]. Jacoby and Lesaux [39] also found that early childhood educators believed young learners’ language proficiency could not be improved through direct language teaching but instead resulted as a natural consequence of young learners’ interactions with each other during the completion of classroom activities.

Such beliefs can be encouraged or influenced by teacher education. Previous studies found an increase in the novice teachers’ efficacy beliefs after receiving teacher training [37,44,45]; after the training, they were more willing to try out new teaching techniques that were introduced in training [37]. Malmberg and Hagger [44] claimed that such changes in teacher beliefs after receiving teacher education are usually related to experiential learning activities such as microteaching in classrooms; teacher education usually has a “meaningful and positive impact” ([44], p. 679). However, this is not to say that all teacher education will positively impact pre-service teachers; if the teacher education is not delivered in a supportive manner, there is the potential risk of a reduction in teaching confidence [46].

### 2.4. Gaps between Pre-Service Teacher Beliefs and Practice

Pre-service teachers’ classroom teaching can be affected by factors originating within or adjacent to teacher education programs [47,48,49]. For example, teacher education programs may use curricula closely tied to teaching practice opportunities; this combination can be helpful in allowing pre-service teachers to put in practice what was taught in the classroom. Such endeavors will only be useful if the pre-service teachers consider the education program as coherent. Canrinus et al. [48] defined education program coherence as a clear vision for pre-service teachers to provide good teaching through interactive idea sharing opportunities in their teacher education classes. Teacher educators’ supervising practicums should ensure they are instilling into pre-service teachers not just “a set of behaviors but a way of being” ([47], p. 228). Therefore, a curriculum composed of meaningful class activities can support the pre-service teachers’ teaching practice and their professional development. Providing pre-service teachers access to meaningful teaching materials [42], and encouraging reflection on teaching through the review of recorded microteaching lessons can lead to pre-service teacher satisfaction [47]. For example, Güven and Çakir [37] found that pre-service primary English teachers’ self-efficacy beliefs changed after taking a course that trained them in how to teach young learners.

Numerous studies have investigated the relationship between teacher beliefs and practices [47,50,51,52,53,54,55]. While many studies have found a reflection of teachers’ beliefs in their teaching, some studies have also found a gap between the two [53,56,57]. For example, Şendil and Erden [53] found that pre-service teachers who believed teachers should be facilitators or guides for young learners were not found to take on the role of a facilitator or guide in their teaching of young learners. Moreover, the researchers found pre-service teachers that claimed “children require the teacher only during activities, not during play time” ([53], p. 627) were found to be actively involved with the children during play time. Furthermore, the pre-service teachers believed play and learning were separate but were found to incorporate teaching activities into children’s play. The gaps between teacher beliefs and practice have been influenced by the teachers’ educational background, teacher education, social norms, and cultural conditions [53,58,59,60].

Educators have attempted to bridge the divide between beliefs and practice by incorporating practical teaching components into their education courses [47,53]. As one example, Sailors et al. [47] provided an experimental group of teachers with teacher education in materials development, workshopping, and directive coaching. The experimental group teachers showed marked positive changes in their beliefs after the teacher education, especially in terms of their self-efficacy of teaching and their beliefs about and their level of comfort in teaching language. However, when their actual teaching was compared to that of the control group teachers that did not receive teacher education, the researchers found no differences in classroom practices. Thus, the ability for teacher education to affect not only beliefs but the manifestation of those beliefs is questionable [53,61].

## 3. Methodology

The purpose of the current study was to explore the beliefs of pre-service pre-primary English teachers in the Macau Special Administrative Region of China and to examine whether these beliefs influenced their pedagogical practices inside the classroom during microteaching activities with their peers. The purpose of this study was accomplished by conducting a case study; this allowed the authors to observe the pre-service teachers’ beliefs through the pedagogical decisions they made during peer microteaching. As cases are bounded by time and activity [62], the authors collected data in the form of written reflections and peer microteaching from the pre-service pre-primary teachers while they were enrolled in a teacher education course over a four-month period of time. The research reported in this paper is only one part of a much larger study conducted on the beliefs of pre-service pre-primary English teachers and teacher educators. The methodology was approved by the ethics review board of the University of Macau under number SSHRE19-APP071-FED.

### 3.1. Participants in a Pre-Service Teacher Education Course

Seventy-five third-year pre-service pre-primary education majors enrolled in an English language learning in early childhood course were recruited as participants in the study. They were all female and between the ages of 20 and 21. The 16-week 3-credit course aimed to prepare students to teach English to pre-primary pupils who learn English as a second or foreign language, emphasizing the practice of various language activities. In addition, students learned to design, adopt, and adapt different teaching materials and teaching aids to plan English lessons for pre-primary pupils. At the end of the course, the education students should have gained an understanding of various language teaching approaches and their underlying theories. Although less emphasized, the course also trained students in classroom English, lesson planning, and teaching pronunciation. Course content was delivered through lecturing, and students were required to complete weekly readings, engage in peer microteaching, reflect on their beliefs about teaching English to pre-primary level learners in Macau, provide oral feedback to their peers about their microteaching, and take a final exam.

The majority of class sessions were devoted to the discussion (pair, group, and class) of assigned readings, microteaching, and providing peer and teacher feedback on the microteaching. To aid in the successful integration of theory and teaching approaches into discussions and lesson planning, some class sessions were also reserved for teacher-led lecturing. Presentation slides accompanied the lectures. The assigned readings and lecturing were intended to enhance the understanding of theory, teaching approaches, and related concepts. Students were required to be active listeners during lectures and active readers when reading the assigned readings; they were asked to take notes during the lectures and on the reading materials covered. Prior to microteaching, the pre-service teachers wrote very short lesson plans that included sections on the target students, rationale, goal(s), learning objective(s), materials, assessment, and procedures. After engaging in microteaching, the pre-service teachers received written feedback from their teacher educator, and they were encouraged to provide oral feedback to their peers. The teacher educator required the pre-service teachers to offer oral feedback to their peers on both areas for improvement and areas that were handled well. These learning activities were aimed at developing the pre-service teachers’ teaching philosophies for teaching pre-primary pupils EFL in the Macau SAR. The required course readings were taken from eight sources (mostly teacher education textbooks) that focused on the teaching of English to young learners. The topics covered included: children learning a foreign language, children learning language through tasks and activities, children learning spoken language and pronunciation, children learning words, children learning through stories, classroom English and classroom management for teaching children; creating, adapting, and evaluating activities for young language learners; syllabus and lesson planning for young learners, and young learner assessment.

### 3.2. Data Collection

Data was collected from the participants twice during the 16-week course. The first data collection occurred at week 9 when the participants were asked to individually write a reflection on their beliefs about the teaching of English to kindergarten students in the Macau SAR. The participants were allowed to write their reflections in English or Cantonese (their native language); however, all of the participants wrote in English. The 75 written reflections totaled 34,692 running words (*M*_words_ = 463, *SD*_words_ = 230). These handwritten reflections were digitized prior to data analysis. The second data collection occurred at weeks 10 and 11 when the participants engaged in 5–10 min in-class peer microteaching activities (the total time equaled 618 min, *M*_minutes_ = 8; *SD*_minutes_ = 2). The microteaching was video recorded, and these 75 recordings were then transcribed verbatim. The transcription resulted in 57,107 running words (*M*_words_ = 761, *SD*_words_ = 252).

### 3.3. Data Analysis

#### 3.3.1. Data Management

*NVivo 12* was employed to support qualitative data management for this case study. *NVivo 12* provided three major benefits over manual pen-and-paper coding procedures [63]. First, the reliability of the findings was increased by reducing the potentials for human error. Second, *NVivo 12* enhanced the analysis by allowing for easy coding, tracking of codes, and comparison of coding completed by different coders [64]. Third, digitalized categorization of different codes increased the trustworthiness of the findings.

#### 3.3.2. Qualitative Content Analysis

Five qualitative content analysis (QCA) procedures outlined by Schreier [65] were used to systematically code the textual data (i.e., the written reflections and the transcriptions of the microteaching lessons) obtained from the participants. First, the authors became familiar with the data by reading through the written reflections and the microteaching transcripts three times. Once the authors had gained a holistic view of the data, a coding frame was selected for use in completing the QCA. As the current study was part of a much larger study on pre-service pre-primary English teacher and teacher educator beliefs, a concept-driven deductive approach was used to select a coding frame. This sixteen-category coding frame was previously developed by the authors for use with a different yet similar dataset obtained from data collection that involved participants with the same educational background and learning goals [66]. Second, the subcategories were mutually exclusive and based on concepts found in the existing teacher beliefs literature. Third, two coders used the coding frame to independently code the data (see Section 3.3.3). Fourth, the second round of coding was carried out to enhance the trustworthiness and reliability of the coding. Fifth, all the authors scrutinized the results of the QCA to compare the coding results of the written reflections and the microteaching transcripts.

After the participants’ written reflections and microteaching transcripts had been coded (see Section 3.3.3), the two coders independently tallied the number of participants that held the beliefs and the number that practiced the beliefs (see Table 1). These two totals were based on the coding of the written reflections and the microteaching transcripts. After these totals were calculated, the coders then independently calculated the proportion of the participants who held the beliefs and also practiced the beliefs. This was done by dividing the number of participants that practiced the belief by the number of participants that held the belief.

#### 3.3.3. Reliability and Validity of the Coding Process

Four strategies were used to enhance the reliability of the results of this case study. First, prior to coding, an external expert (i.e., a professor of curriculum and instruction) was invited to assess the validity of the coding frame [65]. The expert confirmed that the coding frame used in this study was valid, as it adequately represented the targeted belief themes. Next, the QCA was completed by two different coders (the third and sixth authors). Each coder was unaware of the other’s coding during the coding process (i.e., blind coding). After the first round of coding, the two coders met to compare and discuss their independent coding. In addition to blind coding, double-coding was also used by having each of the coder’s code the same data twice, two weeks apart. Coding performed on the written reflections was completed at the sentence level. Coding performed on the microteaching transcripts was performed holistically, requiring the coders to examine the transcripts for examples of teaching that illustrated the definitions of the belief themes (see Table 1). For example, if a pre-service teacher engaged in observation, testing, or interviewing in order to understand the strengths or weaknesses of the students, then the microteaching transcript for that participant was coded as “beliefs about assessment”. For confirmation, the coders often referenced the microteaching videos when coding the microteaching transcripts. The coefficient of agreement for the second round of coding was above 99%, indicating high reliability [67].

**Table 1:** Pre-service pre-primary english teacher beliefs and practices.

*Theme*	*Description of the Theme*	*Number of Participants That Held the Belief*	*Number of Participants That Practiced the Belief*	*Percentage of Participants that Held the Belief That also Practiced the Belief*
*Beliefs about Classroom Practices*	*refer to teachers’ beliefs regarding “[c]lassroom practice, as a process, involv[ing] multiple agents and their interactions within the classroom as a system. The process can be manifested in diverse formats and structures, and its effectiveness can be influenced by numerous factors both internal and external to the classroom” ([68], p. 489).*	*56*	*54*	*96.43%*
*Beliefs about Lesson Planning*	*refer to teachers’ beliefs about “objectives, procedures, and materials for…learning activit[ies]” ([69], p. 137).*	*33*	*28*	*84.85%*
*Beliefs about EFL Learners and EFL Learning*	*refer to teachers’ conceptions of what it means to be English learners whose first language is not English and how they should learn English language skills in a non-English-speaking community ([69], p. 73).*	*43*	*36*	*83.72%*
*Beliefs about the Goals of Language Teaching*	*are teachers’ beliefs regarding the “creat[ion of] skillful L2 users with all their extra attributes, not shadows of native speakers” ([70], p. 51).*	*11*	*6*	*54.55%*
*Beliefs about Assessment*	*are those beliefs regarding “the act or process of gathering data in order to better understand the strengths and weaknesses of student learning, as by observation, testing, interviews,” and other techniques ([69], p. 12).*	*2*	*1*	*50%*
*Beliefs about Teaching*	*refer to “preferred ways of teaching by teachers…[and] these beliefs are broadly classified under…knowledge transmission…or knowledge construction” ([71], p. 164–165).*	*51*	*16*	*31.37%*
*Beliefs about Pedagogical Knowledge*	*refer to those beliefs about “[p]edagogy as the art or science…of being a teacher, involving methods and techniques of teaching predicated on two conceptions of pedagogy: the liberal, emphasizing the autonomy of the child; and the conservative, emphasizing the authority of the teacher” ([72], p. 822).*	*27*	*7*	*25.93%*
*Beliefs about Content*	*are teachers’ beliefs regarding “[k]nowledge of the subject and its organizing structures” ([73], p. 2).*	*10*	*2*	*20%*
*Beliefs about the Role of Teaching*	*“refer to what teachers do in classrooms” [and can be related to] “teacher identity—the ways that teachers think about themselves and their classroom roles” ([74], p. 3).*	*43*	*0*	*0*
*Beliefs about Learning to Teach*	*are teachers’ beliefs regarding “[k]nowing how to learn from classroom teaching experiences. It means planning these experiences in a way that affords learning and then reflecting on the outcomes in order to maximize the benefits that can be gained from the experiences” ([75], p. 206).*	*20*	*0*	*0*
*Beliefs about Microteaching*	*refer to how teachers feel about the “technique in teacher education that uses short, specific episodes of teaching, usually videotaped, for analysis and instruction” ([69], p. 154).*	*17*	*0*	*0*
*Beliefs about the Self*	*are teachers’ beliefs about themselves which emphasize that “…teacher self-efficacy and teacher emotions can be important ways for…language teachers to enhance [their] overall quality” ([76], p. 1400).*	*16*	*0*	*0*
*Beliefs about the Subject*	*are teachers’ beliefs toward “[a]n area of learning and study; discipline” ([69], p. 246).*	*5*	*0*	*0*
*Beliefs about Hearsay*	*are teachers’ beliefs regarding “[w]hat is done or written, as well as to what is spoken” without evidence-based support ([77], p. 192).*	*2*	*0*	*0*
*Beliefs about Self-evaluation*	*are teachers’ beliefs regarding “judging the quality of their work, based on evidence and explicit criteria, for the purpose of doing better work in the future” ([78], p. 1).*	*2*	*0*	*0*
*Beliefs about Schooling*	*are teachers’ beliefs regarding “their own student experiences... [that] guide their interaction with and evaluation of ideas presented in course- and field-based experiences, causing them to accept, modify, or discount those ideas” ([79], p. 132).*	*2*	*0*	*0*

Note: Participant total *N* = 75.

## 4. Results

Analysis of the written reflections revealed 16 themes related to the pre-service pre-primary teachers’ beliefs about teaching EFL to very young learners. Eight of these beliefs were found to be reflected in the teachers’ microteaching with varying degrees. The proportion of the participants that held these eight beliefs (i.e., found within the written reflections) and practiced these beliefs in their microteaching ranged from 20% to 96.43%. These beliefs included beliefs about (1) classroom practices, (2) lesson planning, (3) EFL learners and learning, (4) the goals of language teaching, (5) assessment, (6) teaching, (7) pedagogical knowledge, and (8) content. None of the participants implemented all these beliefs in their microteaching. The remaining eight beliefs were not reflected in the teachers’ microteaching. These consisted of beliefs about (1) the role of teaching, (2) learning to teach, (3) microteaching, (4) the self, (5) the subject, (6) hearsay, (7) self-evaluation, and (8) schooling. Table 1 provides the name of each belief theme, a description of the belief theme, the number of the participants that held a belief categorized under each belief theme, and the number and percentage of the participants that practiced a belief categorized under each theme.

### 4.1. Pre-Service Pre-Primary Teachers’ Beliefs Reflected in Their Microteaching

#### 4.1.1. Beliefs about Classroom Practices

The most salient beliefs practiced by the participants were their beliefs about classroom practices. Classroom practices were defined by Li and Oliverira ([68], p. 489) as “involv[ing] multiple agents and their interactions within the classroom as a system. The process can be manifested in diverse formats and structures, and its effectiveness can be influenced by numerous factors both internal and external to the classroom”. In the reflections, the participants described an ideal early childhood language classroom as interactive and filled with various types of activities that can ease young EFL learners’ anxiety while motivating them to become more engaged in learning. They believed classroom activities should include singing songs, telling stories, and playing games centered on different topics. For example, Participant 4 explained how she planned to use stories and games to teach:

[Stories and games can] attract students to study the language. Both can make students feel interest[ed] and [become] willing to join in. We can change the content of game[s] and use them in other lesson[s]…Stories can develop children’s formal language use, including vocabulary, phrases, grammar, listening, and oral skills...[while] games [can be used to] focus on phrases or single words.

During the microteaching, Participant 4 used a Winnie the Pooh game that incorporated singing to teach weather vocabulary.

Participant 4:…Now I want to play a game with you. This game [is] call[ed]—weather reporter. Winnie the Pooh and his friend want to go outside, but they don’t know how’s the weather. And [can] anyone help them?

Student A: Yes.

Participant 4: Ok. [Does] anyone want to help them? Yes. You pick a doll [from] this bag. Ok, find his house. [Participant 4 points to a poster with different characters and their homes with different weather. The student must match the doll pulled from the bag to the picture and then tell the weather.] How’s the weather?

Student A: Windy.

Participant 4: Ok, let me sing [it] again. How’s [the] weather?

Student A: Windy. It’s windy today.

Participant 4: Wow, you did a great job. Please go back to your seat. And then, who?

Students: Me!

Participant 4: Ok! One, two, three, go! Lucy! Wow, Jessica! Go find his house! Sing the song! How’s the weather?

Student B: Sunny!

Participant 4: How’s the weather?

Students: Sunny!

#### 4.1.2. Beliefs about Lesson Planning

Harris and Hodges [69] defined beliefs about lesson planning as the “objectives, procedures, and materials for…learning activit[ies]”. In general, a lesson plan should include a description of the lesson, goals and/or objectives, target students, teaching materials, and procedures. Among the thirty-three participants who expressed beliefs about lesson planning, twenty-eight of them illustrated this in their microteaching. For example, Participant 62 explained how teachers should design lessons in which the children “learn by doing” and that language goals should require learners to use language related to their daily life, such as “the actions they do after waking up in the morning”. This belief was also shown in Participant 62’s microteaching.

Participant 62: Good morning, everybody.

Students: Good morning.

Participant 62: OK. Before we start today, I want to talk to you about what we going to do. Today we are talking about things that we do in the morning. OK? After we wake up in the morning, OK?... OK. So, this is wake up. *[Participant 62 shows a picture of a girl waking up.]* First, could you come [to] the front please? Can you do [the action] with me together? *[The student and Participant 62 stretch as if they had just woken up.]*

#### 4.1.3. Beliefs about EFL Learners and Learning

Thirty-six of the participants mentioned their beliefs about EFL learners and EFL learning in their written reflections. They wrote about their future target students by describing their ages, mother tongue, and expected English proficiency. They also explained how the age of the learners required creative approaches for teaching language including designing lessons that were child-centered and activity-oriented. For example, Participant 9 believed that young learners in Macau should be taught English using the English language as long as a teacher offers proper scaffolding:

[Young learners] can be taught English as a second language using English. We know that like ESL [English as a second language] learners, EFL learners in Macau will have many chances to speak or listen [to] English outside the class, such as watching TV [or when] shopping. So, they will notice and want to learn that language. As for the[ir] teacher[s], we need to [provide learners] exposure to English through games and tasks. Teachers can be guides-ensuring students reach their potential by develop[ing] their ZPD [zone of proximal development] through the use of scaffolding.

During the microteaching, Participant 9 considered the target students’ contextual background and their current ZPD. During the microteaching, she mentioned that the students already knew the order and names of the 26 letters. Below is an excerpt from the microteaching where she builds on this previous knowledge mentioned in her lesson plan to have students increase their listening fluency in order to understand the names and sounds of the letters through involvement in game play.

Participant 9: Yes. Today I want to play a game with you. I will start [some] music [and]…when I start the music, you need to follow me [to] walk around the classroom. Can you see all over the classroom there are some letters?

Students: Yes.

Participant 9:…listen to my instruction, and I will say “Can you see a letter B?” and then you need to quickly find the letter before the music stops. Understand?

Students: Yes.

Participant 9: Really?

Student A: Should…should I bring the letter back?

Participant 9: Yes, bring it to me and show the letter to me. Ok? So, are you ready?

Students: Yes.

#### 4.1.4. Beliefs about the Goals of Language Teaching

The beliefs about the goals of language teaching refer to the beliefs about the “creat[ion of] skillful L2 users with all their extra attributes, not shadows of native speakers” ([70], p. 51). We found six participants that wrote reflections regarding beliefs about the goals of language teaching that were evident in their microteaching. The participants emphasized that teachers should always keep their teaching and language learning goals in mind by setting goals that they can guide students in reaching step by step. In addition, some participants mentioned that due to the age and cognitive development stage of very young EFL learners, they were more willing to learn English if taught in an interesting manner in a vivid classroom environment. These participants set goals for their microteaching that incorporated interactive or curiosity-encouraging elements. For example, Participant 11 stated during her microteaching that she wanted the “...students [to] know different animals [which] can give us different products for use in daily life”. She made the students aware of the expected learning outcomes and also got them involved in the lesson through a curiosity-arousing game that required the learners to listen to sounds and guess the names of animals. Then the learners could confirm the guesses by removing small animal figures from a cloth bag. She then sang a verse of a song incorporating each animal’s name and the product it provided. Specifically, Participant 11 related the language learning goal of learning about “farm animals” to those animals that provided products, such as milk from cows or wool from sheep, used by the children in their daily lives. Participant 72 and others stated in their written reflections that teachers should be “using stories and games [that] have a clear goal. That is when students will learn language [from] the teaching”. During the microteaching, Participant 72 also stated the goals in preparation for the execution of the activity:

Participant 72: Today we are going to play a game–a very fun game. After playing this game, I believe that most of you will remember all the names of the fruits. Ok? And here are five flash cards in my hand. And all of you should have a flash card. So, I will just hand them out. Dara, Ruth, Suzie, and Tricia. So, hey, just look at me. What am I holding in my hand?

Students: Apple.

Participant 72: Yes, very good. It’s [an] apple. So, I am holding an apple in my hand. I am going to say this: “apple down, apple down, apple down, then orange down”. So, the student who is holding an orange needs to follow me and do what I did. You can say any…[of] the five fruits, ok? You can also say banana, or you can say lemon, or you can say pear. So, can you guys do it?

Students: Yes.

#### 4.1.5. Beliefs about Assessment

Teachers’ beliefs about assessment regard beliefs about “the act or process of gathering data in order to better understand the strengths and weaknesses of student learning, as by observation, testing, interviews and other techniques” ([69], p. 12). Altogether, there were two participants that discussed their beliefs about assessment in their written reflections; however, only one participant exhibited these beliefs in her microteaching.

The two participants that wrote about their assessment beliefs felt that there were two types of assessments. The first type of assessment occurred right after teaching and was used by a teacher to assess learning of “just taught material”. Another type of assessment was used to assess whether what students had previously learned had been retained; this type of assessment should be provided at a later date. They referred to these as “immediate assessment” and “delayed assessment,” and those assessments could be given as games the young learners would play. More specifically, Participant 54 wrote about how assessments need to be administered in a clear way, or learners may be confused:

The number of assessments can [be many]…but the assessment in [a] game may not [be] efficient or effective [if] I [do] not provide a clear instruction of the game rules. The kids may misunderstand the rules.

During the microteaching she used direct questioning that required students to verbally provide answers. This was a form of what was referred to as “delayed assessment,” as she was confirming their knowledge of previously taught color words.

Participant 54:…Who can tell me the colors that we learned last time? What is this? *[Participant 54 shows a card with a color square.]*

Student A: This is red.

Participant 54: OK. What color is it?

Student B: It is orange.

Participant 54: And then what color is it?

Student C: It is yellow.

Participant 54: Yes. And the last one, what color is it?

Student D: It is green.

Participant 54: Great!

#### 4.1.6. Beliefs about Teaching

“Beliefs about teaching…referred to the preferred ways of teaching by teachers” ([71], p.164). Sixteen participants mentioned their beliefs about teaching in their written reflections, and these beliefs were also shown in their microteaching. Some participants believed that good teaching needs to balance teacher-centered and student-centered methods. Some also wrote that exaggerated body movements are needed when teaching to convey meaning to young learners. They also thought pre-teaching and demonstrations were needed to successfully teach an EFL lesson to very young learners. For example, Participant 67 shared:

Preparation include[s] pre-teaching of language items and vocabulary. [These] prepare the learners to be able to complete a core activity successfully.

When teaching left, right, forward, and backward, Participant 67 first pre-taught these four words using pictures and her body movements before asking the learners to perform the same body movements when she sang a song that included the targeted words.

Participant 67:…Ok, I will sing a song. When I sing the word forward, you should do the forward action like this *[Participant 67 performs the action]*: step one, step two, and clap your hands twice. Ok, listen, pay attention. *[She sings the song.]* “The teacher says move forward, the teacher says move right, the teacher says move backward, the teacher says move left”. Very good.

Other participants mentioned that effective teaching included activities that gradually increased in difficulty, or that teaching incorporating the native language (Cantonese) should be used to reduce difficulty. For example, Participant 4 believed that Cantonese should be used before using English when teaching. She considered that teaching in this way could “create a positive environment for learners” by “scaffolding the kids when they cannot understand well”. During the microteaching lesson about “a rainy day,” she moved between the two languages. She also used Cantonese to first introduce the language content she was going to teach in English before switching back to English to teach that language content.

Participant 4: Can anyone tell me what is your feeling on [a] rainy day?

Student A: Water.

Teacher: Yes. On [a] rainy you may get wet! 它會令到我們濕了。然後呢，我們需要帶什麼？雨傘！然後呢，要穿雨衣。還有，雨鞋。 [It makes us wet. And then? What do we need to bring? Umbrella! And then? We need to wear raincoats. What else? Rain boots.] Please read after me. Rainy.

Students: Rainy.

Participant 4: Rainy.

Students: Rainy.

#### 4.1.7. Beliefs about Pedagogical Knowledge

Participants’ beliefs about pedagogical knowledge refer to those beliefs about “being a teacher, involving methods and techniques of teaching predicated on two conceptions of pedagogy: the liberal, emphasizing the autonomy of the child; and the conservative, emphasizing the authority of the teacher” ([72], p. 822). Seven participants were found to put the pedagogies and approaches they described in their written reflections to practice in their microteaching. The most commonly practiced pedagogy was Total Physical Response (TPR). TPR requires “learners to respond physically to imperatives given by the teacher, who has contrived these imperatives in such a way as to cover the linguistic items that he or she wishes to teach” ([80], p. 270). TPR encourages the students’ involvement in language through body movements that children intuitively react to as play, stimulating an intrinsic interest in English language learning. Most often a teacher will demonstrate TPR to the students before requiring actions or reactions to given commands. These actions become increasingly more difficult. Participant 67 explained TPR in her written reflection as:

A teaching method [using] a lot of physical movements and experiences to assess teaching and learning...Young learners listen and do actions and follow instructions. While they feel energetic [they also feel] relaxed…they are learning and absorbing language.

Section 4.1.6. provides an excerpt from Participant 67’s microteaching lesson where she applied TPR to teach direction words.

#### 4.1.8. Beliefs about Content

Two participants held beliefs about content and were found to demonstrate these beliefs in their microteaching. Beliefs about content refers to “[k]nowledge of the subject and its organizing structures” ([73], p. 2). These participants said considering the difficulty and relevance of content was important. They felt it was important to teach content that was related to students’ daily lives and was not too difficult. For example, they might teach weather words that were related to the type of weather the students would actually experience, and they would not ask students to do things that were beyond their ability level, such as to read or write out words. The participants were also very concerned about the accuracy of the language content they taught the young learners. Participant 70 explained that a “teacher must teach and model correct language content. If the teacher does not do that, student[s] may learn it wrong”. During her microteaching, she taught students language that could be used to describe their bodies, which she considered useful and relevant to three-year-old learners. She also noted that since the students were just learning English, she used both the mother tongue (Cantonese) and English in her lesson:

Participant 70: OK now we are going to learn new phrases today. 我們今天要學習一個新的詞組叫 [We are going to learn a new word today, it is] body. Repeat. Body.

Students: Body.

Participant 70: Again.

Student A: Body.

Participant 70: What does it mean?

Student B: 全身 [body].

Participant 70: 對表示我們的全身。[Right, this means our body.] Body, OK? Body.

Students: Body.

Teacher: Good. And now we are going to do an exercise. I will ask you some questions and then you answer me. When I say body, everyone does this action, OK? *[Participant 70 does an action, and the students copy.]*

### 4.2. Pre-Service Pre-Primary Teachers’ Beliefs Not Reflected in Their Microteaching

#### 4.2.1. Beliefs about the Role of Teaching

Defined by Keiler [74] as “what teachers do in classrooms,” beliefs about the role of teaching were touched upon by 43 participants in their written reflections. They discussed their roles as teachers in the classroom, reflecting different opinions on their identities. Many participants described themselves as guides or planners. For example, Participant 2 explained:

A teacher is a guide [and] a planner...[Teachers as guides] lead children through different activities by giv[ing] them instructions. For example, when introducing a new game, the teacher can scaffold the learners to understand the directions. [A teacher can be a] planner [when] the teacher design[s] or picks suitable activities at [the] right difficulty level, [for the] right social context, [containing the] right values and [that helps] achieve the goal. 

#### 4.2.2. Beliefs about Learning to Teach

Beliefs about learning to teach are teachers’ beliefs regarding “[k]nowing how to learn from classroom teaching experiences. It means planning these experiences in a way that affords learning and then reflecting on the outcomes in order to maximize the benefits that can be gained from the experiences” ([75], p. 206). Twenty participants shared their beliefs about learning to teach acquired from their part-time teaching experiences. For example, Participant 65 shared:

I [was] a[n] English tutor for 3–5-year-old kids whose first language is Cantonese. At that time, I tried many methods to teach vocabulary in English, but I found it [was] hard for them; then I realized I shouldn’t force them to learn language so quick[ly]. Then, I tr[ied] to motivate them and found many jokes and even magic [tricks] to make them interest[ed in the lessons]. Then they start[ed] to have passion [and] focused on my teaching. That’s when I noticed what I should do is to first motivate kids, and then be their guide [who can] lead them to learn English step by little step.

#### 4.2.3. Beliefs about Microteaching

Teachers’ beliefs about microteaching refer to how teachers feel about the “technique in teacher education that uses short, specific episodes of teaching, usually videotaped, for analysis and instruction” ([69], p. 154). It was found that seventeen participants mentioned their beliefs about microteaching in their written reflections. A large proportion of these participants mentioned that they were looking forward to this experience and felt it could help them to become qualified in-service teachers. For example, Participant 27 shared:

Microteaching [provides] a good practice for us to try [things] and share ideas about planning activities for kindergarten [students]. It enables us to learn from one another and inspires some new thoughts.

#### 4.2.4. Beliefs about Self

Beliefs about self refer to the participants’ beliefs about themselves, emphasizing that “…teacher self-efficacy and teacher emotions can be important ways for us language teachers to enhance our overall quality” ([76], p. 1400). Sixteen participants held beliefs about themselves. Participant 45 shared several insecurities. She shared that, “It is a bit hard for me to immediately speak English without having any script...Tak[ing] care of the children’s feelings and meeting my teaching objectives is too hard a job for me”. Other teachers, such as Participant 49, also shared reservations about their English language proficiency:

This will be my first time [to] use English to teach a lesson. I have no confidence in my oral English ability, so l am too nervous. It makes my speed of speech too fast, [so] I need to improve my [oral] English [fluency].

#### 4.2.5. Beliefs about the Subject

Beliefs about the subject are the teachers’ beliefs about “[a]n area of learning and study; discipline” ([69], p. 246). In this study, the subject refers to the English language taught in the Macau kindergarten classroom. Five participants mentioned their beliefs about English in their written reflections. Most of these participants admitted the importance of keeping English as a part of early childhood curriculum in Macau. For example, Participant 24 stated that “English is a very important language. People from many countries speak English”. Participant 58 also agreed that “Nowadays, English is one of the [most] important language[s] around us”.

#### 4.2.6. Beliefs about Hearsay

Beliefs about hearsay are the participants’ beliefs regarding “[w]hat is done or written, as well as…what is spoken” without evidence-based support ([77], p. 192). Two participants in this study stated beliefs that were based on hearsay. For example, Participant 34 mentioned that she believed certain aspects about learning a second language that were told to her by someone else. Participant 27 shared some advice that someone else gave her in the past about how young children learn a second language and she also believed what they told her.

#### 4.2.7. Beliefs about Self-Evaluation

Beliefs about self-evaluation are teachers’ beliefs regarding “judging the quality of their work, based on evidence and explicit criteria, for the purpose of doing better work in the future” ([78], p. 1). Two participants shared their beliefs about self-evaluation in their written reflections. Participant 28, for example, provided a thorough self-assessment of what she planned for the microteaching:

When I think about my microteaching, I [get] very nervous. My chosen theme is birthday…but I think I can’t show the theme very clear[ly]…I think that I can do a good job [and] not [be] too fast or slow. And I [have] done a good job in teaching the birthday song in the past…In terms of teaching aids, birthday scenes, birthday cakes, and candles can be used…[and] I can arrange the classroom like [a] birthday party so that children can feel the birthday atmosphere [is] more realistic... I can think of more ways to interact with students, giv[ing] students more opportunities to speak and act, [and I can] try to avoid speaking too much…The tone of my speech should be lively and [I should] try to speak as smoothly as possible.

#### 4.2.8. Beliefs about Schooling

Beliefs about schooling refer to the participants’ beliefs regarding “their own student experiences…[that] guide their interaction with and evaluation of ideas presented in course- and field-based experiences, causing them to accept, modify, or discount those ideas” ([79], p. 132). Only two participants shared their beliefs about the influence of their schooling. For example, Participant 33 explained how the medium of instruction in her school might have influenced her future teaching ability, since she was taught in a school that used Cantonese and not English as the language of instruction. She felt because of her lack of experience learning in English, it would be difficult for her to teach in English. She felt her English was “weaker” and felt “the more [she] fears, the more [she would] have to face”.

## 5. Discussion

The findings showed 16 different beliefs about various aspects of teaching English to very young learners held by the pre-service pre-primary teachers after participating in an English teaching methodology course in a bachelor of pre-primary education program. Eight of the beliefs were reflected in their classroom microteaching in varying degrees, while the remaining eight beliefs were not implemented in their practices. These differences can be explained in relation to the course content and activities, the student teachers’ background, and the sociocultural factors that may influence the teachers’ beliefs and practices.

Most notably, beliefs about (1) classroom practices, (2) lesson planning, and (3) EFL learners and learning were the most predominant among the participants. Also, these beliefs were reflected quite well in the teachers’ classroom microteaching (83–96%). This could be due to the prevalence of these aspects in the course. Specifically, the pre-service teachers spent a large amount of time learning how to deal with classroom practices such as setting up learning tasks, giving instructions, and classroom management. They also completed a significant amount of practice designing lesson plans for microteaching. While discussing and repeating these two aspects of classroom practice, the student teachers were told to keep in mind the characteristics and socio-cultural backgrounds of their target learners, as well as their learning goal. This might have led to the development of teachers’ beliefs about EFL learners and learning. It should be noted that, within the bachelor of pre-primary education program taken under study, this course was the only one that specifically focused on the methodology of teaching EFL. Therefore, it is not surprising to observe that these three beliefs were held by the majority of the participants and were largely implemented in their practice. It should be mentioned that the course was successful in developing the pre-service teachers’ beliefs and practices regarding these most important aspects of EFL teaching methodology. These findings support Reynolds et al.’s [66] study with pre-service pre-primary teachers in Macau. Also, these findings are generally in line with the literature in L2 teacher education and professional development [33,44,81,82,83]. Busch [81], for example, argued that although teachers’ beliefs are difficult to change, educational courses that provide pre-service teachers with sufficient opportunities to practice their new knowledge gained in the course could affect teachers’ cognitive changes. Ha and Murray’s [82] study supported this argument, further arguing that even with experienced teachers and complex aspects of teacher learning, experiential learning and reflective activities could help teachers change their beliefs.

What is more notable about these findings is the pre-service teachers’ beliefs observable in their microteaching. Previous studies in teacher education research looked mainly at the development of teachers’ beliefs. The current study showed that the teachers could implement some of their beliefs about important aspects of English language teaching to very young learners in their microteaching. Specifically, the development of beliefs about classroom practices, as well as beliefs about EFL learners and learning, are particularly important for the participants in the current study. As opposed to previous studies regarding EFL pre-service teachers’ beliefs about teaching practices where the teachers were exposed to various courses of EFL-related subjects and EFL teaching, the participants in the current study were only exposed to EFL teaching methodology in this course because their bachelor program focused on early childhood education and not language education. Therefore, these beliefs and practices would be very significant for them, helping them prepare for the subsequent practicum experience and for real teaching in the workplace.

The second set of beliefs that were relatively evident in the pre-service teachers’ practices included beliefs about (1) the goals of language learning, (2) assessment, (3) teaching, (4) pedagogical knowledge, and (5) content (from 20% to 55% match observed in microteaching). These mismatches seem to be natural because not all beliefs could be reflected in the classroom practices due to the complex nature of the teaching. Similar findings were also reported in the literature in that teachers’ beliefs and practices are not always congruent [31,32,53,56,84], especially studies with pre-service teachers [85]. This may be due to the fact that the pre-service teachers did not have sufficient time and opportunities to turn their theoretical knowledge and beliefs into practice. The findings that the teachers held various beliefs about different aspects of English language teaching and learning (rather than focusing on a particular aspect) differed from some studies on EFL teacher education where pre-service teachers were reported to focus primarily on developing students’ English proficiency [3]. The teachers in this current study reported more concerns about how to teach (e.g., how to create an enjoyable, lively classroom atmosphere) rather than what to teach (e.g., content). This may be due to the influence of the teachers’ conception of the overall objective of early childhood education, that is, their main role is to help young learners have fun while learning via learning by playing.

Regarding the remaining reported beliefs, there were no observable practices related to any of these beliefs. These included beliefs about (1) the role of teaching, (2) learning to teach, (3) microteaching, (4) the self, (5) the subject, (6) hearsay, (7) self-assessment, and (8) schooling. This result appears to be surprising. However, this may be due to the fact that the microteaching lasted from only five to ten minutes, which did not allow the practice of these activities to occur. Secondly, some of these beliefs may not be easily shown in practice, even when the teachers were observed for a more extended period of time. For example, beliefs about schooling (i.e., their experiences as students) may only be revealed in practice in the end-of-term teaching sessions where the teachers spend more time giving students feedback concerning a period of study. It should be noted that the absence in the microteaching of aspects related to these beliefs does not mean that the teachers or the course failed. The development of the teachers’ cognitive aspects, such as teachers’ beliefs, knowledge, and understanding, were particularly important because such development could help pre-service teachers reflect on their on-going teacher learning process during the course study, the practicum, and the initial period of formal teaching. Such processes would help teachers to become reflective practitioners [86] and subsequently, effective teachers.

## 6. Limitations and Future Directions

This study has several limitations that need to be mentioned as a caveat to readers as they interpret the findings. Firstly, the microteaching lessons lasted from five to ten minutes each; therefore, there were not sufficient opportunities for the teachers to illustrate all the teaching aspects related to the 16 belief themes. Firstly, this limitation was due to the fact that the current study was conducted with two intact classes in a bachelor of pre-primary education program, and the duration of microteaching was fixed according to the course guidelines. Secondly, the study was qualitative in nature, but it involved a large number of participants, which limited the study data to observations and written reflections. Future studies could employ stimulated recall interviews following participants’ microteaching to take an emic view and gain more insights into the matches or mismatches between teachers’ beliefs and practices. Thirdly, another possible limitation is that the teachers’ beliefs were captured at a particular moment in time after they had received around two months of English teacher education and engaged in peer teaching. Future studies could take a longitudinal study design to document the trajectory of teachers’ beliefs throughout their teacher education program. For example, future studies could ask student teachers to keep a journal to reflect on their own microteaching, as well as that of their peers, throughout the English language teacher education course.

## 7. Conclusions and Implications

The current study explored the beliefs of pre-service pre-primary teachers about EFL teaching for very young learners and whether such beliefs were reflected in their microteaching. Analyses of written reflections and microteaching by the participants (75 pre-service teachers) showed that the teachers held 16 beliefs about various aspects of teaching EFL to preschool learners, although not all of the teachers held the same beliefs. Some of these beliefs, such as beliefs about classroom practice, lesson planning, and EFL learners and learning, were held by the majority of the teachers and were clearly reflected in their microteaching. However, eight of the beliefs were missing in the teachers’ classroom practices.

This study provided several novel insights into the current literature of teacher education research. Firstly, it was one of the few reported attempts to explore the beliefs and practices of pre-service pre-primary EFL teachers, an underexplored research area. Most of the previous literature focused on either EFL teachers who were pursuing a bachelor’s degree in EFL teaching, or on early childhood teachers in general. The current study showed that a well-designed educational course could equip pre-service teachers to develop various beliefs and practices regarding important areas of EFL teaching. Classroom practice, lesson planning, and appropriate conceptions of EFL learners and learning are perhaps the most important areas of learning for pre-service teachers. The teachers in the present study clearly showed these beliefs in their written reflections and microteaching. This could be seen as a sign of the success of the course, which might be due to the richer exposure to these aspects during the study processes. This suggests that future similar educational courses should emphasize experiential and reflective learning components which can help teachers shape their beliefs and practices regarding these essential areas of teaching in preparation for their practicum and initial formal teaching experiences.

It should be noted that this study was not designed to investigate the match or mismatches between pre-service teachers’ beliefs and practices. Instead, the main purpose was to explore what beliefs the pre-service teachers may have developed during their EFL teaching methodology course, and what aspects of these beliefs may be reflected in their microteaching. The exploratory nature of the study allows us to provide some useful insights into the learning and development of some cognitive aspects and practices related to EFL teaching in a particular context. Apart from the more prominent finding that eight beliefs were reflected in the teachers’ microteaching, the other findings may also have important implications. While it may be due to the fact that the limited microteaching duration did not allow the teachers to implement their beliefs into practice, this finding may also suggest that EFL teacher education course designers should clearly articulate the objectives of the course so that particular activities can be prioritized. By providing future pre-service teachers with sufficient opportunities to reflect on what they learn, the teacher educators will allow them the chance to shape their understanding, knowledge, and beliefs about important aspects of teaching EFL to young learners, motivating them to implement such beliefs in their microteaching. Thus, pre-service teachers will be prepared for their practicum and initial formal teaching.

## Data Availability

The data presented in this study are available on request from the corresponding author. The data are not publicly available due to signed consent of the research participants.

## References

[1] Borg S. (2003). Teacher cognition in language teaching: A review of research on what language teachers think, know, believe, and do. Lang. Teach. Res..

[2] Farrell T.S.C. (2006). ‘The teacher is an octopus’: Uncovering pre-service English language teachers’ prior beliefs through metaphor analysis. Reg. Lang. Cent. J..

[3] Zheng H. (2009). A review of research on EFL pre-service teachers’ beliefs and practices. J. Camb. Stud..

[4] Calderhead J., Berliner D.C., Calfee R.C. (1996). Teachers: Beliefs and Knowledge. Handbook of Educational Psychology.

[5] Mattheoudakis M. (2007). Tracking changes in pre-service EFL teacher beliefs in Greece: A longitudinal study. Teach. Teach. Educ..

[6] Shieh J.-J., Reynolds B.L. (2021). The origin and impact of an ESL teacher’s beliefs on curriculum design. Asia Pac. J. Educ..

[7] Cheung A., Hennebry-Leung M. (2020). Exploring an ESL teachers’ beliefs and practices of teaching literary texts: A case study in Hong Kong. Lang. Teach. Res..

[8] Watzke J.L. (2007). Foreign language pedagogical knowledge: Toward a developmental theory of beginning teacher practices. Mod. Lang. J..

[9] Mann S. (2005). The language teacher’s development. Lang. Teach..

[10] Yang Z. (2018). Chinese Early Childhood English Education from the Perspective of Ecological Language. Proceedings of the 2018 8th International Conference on Management, Education and Information (MEICI 2018).

[11] Liu S., Reynolds B.L., Ha X.V., Ding C. (2021). Professionals as collaborative mentors in early childhood family education. Sustainability.

[12] Moody A. (2008). Macau English: Status, functions and forms. Engl. Today.

[13] Borg S., Al-Busaidi S. (2012). Learner Autonomy: English Language Teachers’ Beliefs and Practices.

[14] Reynolds B.L., Liu S., Milosavljevic M., Ding C., McDonald J.A. (2021). Exploring pre-service pre-primary EFL teacher beliefs about teaching English to very young learners: A Macau case study. Sage Open.

[15] Junqueira L., Kim Y. (2013). Exploring the relationship between training, beliefs, and teachers’ corrective feedback practices: A case study of a novice and an experienced ESL teacher. Can. Mod. Lang. Rev..

[16] Vartuli S. (1999). How early childhood teacher beliefs vary across grade level. Early Child. Res. Q..

[17] Rahimi M., Zhang L.J. (2015). Exploring non-native English-speaking teachers’ cognitions about corrective feedback in teaching English oral communication. System.

[18] Brown J.P. (2017). Teachers’ perspectives of changes in their practice during a technology in mathematics education research project. Teach. Teach. Educ..

[19] Buldu M., Erden F.T. (2017). An investigation of Turkish early childhood teachers’ self-reported beliefs and practices regarding assessment. J. Educ. Future.

[20] Chan W.L. (2016). The discrepancy between teachers’ beliefs and practices: A study of kindergarten teachers in Hong Kong. Teach. Dev..

[21] Bekleyen N. (2011). Can I teach English to children? Turkish pre-service teacher candidates and very young learners. J. Early Child. Teach. Educ..

[22] Betawi A., Jabbar S. (2019). Developmentally appropriate or developmentally inappropriate, that’s the question: Perception of early childhood pre-service teachers at The University of Jordan. Int. J. Adolesc. Youth.

[23] Brown B. (2016). A systems thinking perspective on change processes in a Teacher Professional Development programme. J. Educ..

[24] Le L.T.B., Tran T.T., Tran N.H. (2021). Challenges to STEM education in Vietnamese high school contexts. Heliyon.

[25] Brown C.P., Ku D.H., Barry D.P. (2020). Making sense of instruction within the changed kindergarten: Perspectives from pre-service early childhood educators and teacher educators. J. Early Child. Teach. Educ..

[26] Akaba S., Peters L.E., Liang E., Graves S.B. (2020). Pre-K teachers’ perspectives on factors that influence their experiences through universal Pre-K policy changes in New York City. J. Early Child. Res..

[27] Alacam N., Olgan R. (2019). Pre-service early childhood teachers’ beliefs concerning parent involvement: The predictive impact of their general self-efficacy beliefs and perceived barriers. Education.

[28] Ha X.V., Nguyen L.T. (2021). Targets and sources of oral corrective feedback in English as a foreign language classrooms: Are students’ and teachers’ beliefs aligned?. Front. Psychol..

[29] Ha X.V., Nguyen L.T., Hung B.P. (2021). Oral corrective feedback in English as a foreign language classrooms: A teaching and learning perspective. Heliyon.

[30] Chaaban Y., Du X., Ellili-Cherif M. (2019). Influence of the practicum experience on student-teachers’ beliefs about their role in EFL classrooms. Int. J. Learn. Teach. Educ. Res..

[31] Tran N.G., Ha X.V., Tran N.H. (2021). EFL reformed curriculum in Vietnam: An understanding of teachers’ cognitions and classroom practices. RELC J..

[32] Ha X.V., Tran N.G., Tran N.H. (2021). Teachers’ beliefs and practices regarding assessment in English as a foreign language classrooms in Vietnam. Qual. Rep..

[33] Ha X.V. (2021). Oral Corrective Feedback in Vietnamese EFL Classrooms: Effects of Awareness-Raising Activities on Teachers’ Beliefs and Practices. Ph.D. Thesis.

[34] Altan M.Z. (2012). Pre-service EFL teachers’ beliefs about foreign language learning. Eur. J. Teach. Educ..

[35] Cohen A.D., Dörnyei Z., Schmitt N. (2002). Focus on the Language Learner: Motivation, Styles, and Strategies. An Introduction to Applied Linguistics.

[36] Voss T., Kunter M. (2019). “Reality shock” of beginning teachers? Changes in teacher candidates’ emotional exhaustion and constructivist-oriented beliefs. J. Teach. Educ..

[37] Güven S., Çakir Ö. (2012). A study on primary school English teachers’ self-efficacy beliefs. Egit. Ve Bilim-Educ. Sci..

[38] Brumfit C., Brumfit J., Moon R., Tongue R. (1991). Introduction: Teaching English to children. Teaching English to children.

[39] Jacoby J.W., Lesaux N.K. (2019). Supporting dual language learners in Head Start: Teacher beliefs about teaching priorities and strategies to facilitate English language acquisition. J. Early Child. Teach. Educ..

[40] Nafissi Z., Shafiee Z. (2020). Teachers’ roles in early childhood English language pedagogy: Beliefs of kindergarten English language teachers. J. Early Child. Teach. Educ..

[41] Pupiková E., Gonda D., Páleniková K., Medová J., Kolárová D., Tirpáková A. (2021). How kindergarten teachers assess their own professional competencies. Educ. Sci..

[42] McMahon R., Richmond M.G., Reeves-kazelskis C. (1998). Relationships between kindergarten teachers’ perceptions of literacy acquisition and children’s literacy involvement and classroom materials. J. Educ. Res..

[43] Haznedar B. (2003). The status of functional categories in child second language acquisition: Evidence from the acquisition of CP. Second Lang. Res..

[44] Malmberg L.-E., Hagger H. (2009). Changes in student teachers’ agency beliefs during a teacher education year, and relationships with observed classroom quality, and day-to-day experiences. Br. J. Educ. Psychol..

[45] Woolfolk Hoy A.W., Spero R.B. (2005). Changes in teacher efficacy during the early years of teaching: A comparison of four measures. Teach. Teach. Educ..

[46] Pigge F.L., Marso R.N. (1997). A seven year longitudinal multi-factor assessment of teaching concerns development through preparation and early years of teaching. Teach. Teach. Educ..

[47] Sailors M., Hoffman J.V., Pearson P.D., McClung N., Shin J., Phiri L.M., Saka T. (2014). Supporting change in literacy instruction in Malawi. Read. Res. Q..

[48] Canrinus E.T., Bergem O.K., Klette K., Hammerness K. (2017). Coherent teacher education programmes: Taking a student perspective. J. Curric. Stud..

[49] Goh P.S.C., Canrinus E.T., Wong K.T. (2020). Preservice teachers’ perspectives about coherence in their teacher education program. Educ. Stud..

[50] Cancino M., Durán M., Solorza C. (2020). What learning can do to teaching: Assessing the impact of apprenticeship of observation on pre-service teachers’ beliefs. Engl. Teach. Learn..

[51] Cornett J.W. (1990). Teacher thinking about curriculum and instruction: A case study of a secondary social studies teacher. Theory Res. Soc. Educ..

[52] Pajares M.F. (1992). Teachers’ beliefs and educational research: Cleaning up a messy construct. Rev. Educ. Res..

[53] Şendil Ç.Ö., Erden F.T. (2019). Understanding the gaps between Turkish teachers’ beliefs and practices for dealing with preschoolers’ peer relationship problems. Early Educ. Dev..

[54] Schachter R.E., Spear C.F., Piasta S.B., Justice L.M., Logan J.A.R. (2016). Early childhood educators’ knowledge, beliefs, education, experiences, and children’s language- and literacy-learning opportunities: What is the connection?. Early Child. Res. Q..

[55] Richardson V., Anders P., Tidwel D., Lloyd C. (1991). The relationship between teachers’ beliefs and practices in reading comprehension instruction. Am. Educ. Res. J..

[56] Basturkmen H., Loewen S., Ellis R. (2004). Teachers’ stated beliefs about incidental focus on form and their classroom practices. Appl. Linguist..

[57] Cheung R.H.P. (2012). Teaching for creativity: Examining the beliefs of early childhood teachers and their influence on teaching practices. Aust. J. Early Child..

[58] Kissau S.P., Algozzine B., Yon M. (2012). Similar but different: The beliefs of foreign language teachers. Foreign Lang. Ann..

[59] Raymond A.M. (1997). Inconsistency between a beginning elementary school teacher’s mathematics beliefs and teaching practice. J. Res. Math. Educ..

[60] Shi Q., Zhang S., Lin E. (2014). Relationship of new teachers’ beliefs and instructional practices: Comparisons across four countries. Action Teach. Educ..

[61] Zheng H. (2013). The dynamic interactive relationship between Chinese secondary school EFL teachers’ beliefs and practice. Lang. Learn. J..

[62] Cresswell J.W., Cresswell J.D. (2018). Research Design: Qualitative, Quantitative, and Mixed Methods Approaches.

[63] Creswell J.W. (2019). Educational Research: Planning, Conducting, and Evaluating Quantitative and Qualitative.

[64] Leavy P. (2014). The Oxford Handbook of Qualitative Research.

[65] Schreier M. (2012). Qualitative Content Analysis in Practice.

[66] Reynolds B.L., Liu S., Ha X.V., Zhang X., Ding C. (2021). Pre-service teachers learning to teach English as a foreign language to preschool learners in Macau: A longitudinal study. Front. Psychol..

[67] Miles M.B., Huberman A.M. (1994). Qualitative Data Analysis: An Expanded Sourcebook.

[68] Li Y., Oliveira H., Cho S.J. (2015). Research on Classroom Practice. The Proceedings of the 12th International Congress on Mathematical Education.

[69] Harris T.L., Hodges R.E. (1995). The Literacy Dictionary: The Vocabulary of Reading and Writing.

[70] Cook V. (2013). What are the goals of language teaching?. Iran. J. Lang. Teach. Res..

[71] Teo T., Chai C.S., Hung D., Lee C.B. (2008). Beliefs about teaching and uses of technology among pre-service teachers. Asia Pac. J. Teach. Educ..

[72] Farquhar S., White E.J. (2014). Philosophy and pedagogy of early childhood. Educ. Philos. Theory.

[73] Ball D.L., Thames M.H., Phelps G. (2008). Content knowledge for teaching: What makes it special. J. Teach. Educ..

[74] Keiler L.S. (2018). Teachers’ roles and identities in student-centered classrooms. Int. J. STEM Educ..

[75] Hiebert J., Morris A.K., Glass B. (2003). Learning to learn to teach: An “experiment” model for teaching and teacher preparation in mathematics. J. Math. Teach. Educ..

[76] Xu L. (2012). The role of teachers’ beliefs in the language teaching-learning process. Theory Pract. Lang. Stud..

[77] Falknor J.F. (1940). Silence as Hearsay. Univ. Pa. Law Rev. Am. Law Regist..

[78] Rolheiser C., Ross J.A., Small R.D., Thomas A. (2001). Student Self-Evaluation: What Research Says and What Practice Shows. Plain Talk about Kids.

[79] Schmidt M. (2010). Learning from teaching experience: Dewey’s theory and pre-service teachers’ learning. J. Res. Music. Educ..

[80] Sano M. (1986). How to incorporate total physical response into the English program. ELT J..

[81] Busch D. (2020). Pre-service teacher beliefs about language learning: The second language acquisition course as an agent for change. Lang. Teach. Res..

[82] Ha X.V., Murray J.C. (2021). The impact of a professional development program on EFL teachers’ beliefs about corrective feedback. System.

[83] Vásquez C., Harvey J. (2010). Raising teachers’ awareness about corrective feedback through research replication. Lang. Teach. Res..

[84] Ha X.V., Murray J.C. (2020). Corrective feedback: Beliefs and practices of Vietnamese primary EFL teachers. Lang. Teach. Res..

[85] Kartchava E., Gatbonton E., Ammar A., Trofimovich P. (2020). Oral corrective feedback: Pre-service English as a second language teachers’ beliefs and practices. Lang. Teach. Res..

[86] Farrell T.S. (2015). Reflective Language Teaching: From Research to Practice.

